# The Subcutaneous Injection of Ether

**Published:** 1882-10

**Authors:** 


### THE SUBCUTANEOUS INJECTION OF ETHER.

An editorial in the *Medical News* for August 2, commenting on the fact that the powerful stimulating effect of ether is not generally known, states the conditions under which it may, with advantage, be applied. Thus it is useful in adynamic pneumonia, in fevers when failure of the vital powers is threatened, in the puerperal state, and in cases of thrombosis of important vessels. Besides, its use as a stimulant in conditions of depression it has important applications as a hypnotic and local anodyne. It is useful in cerebral excitement and wakefulness, accompanied by arterial depression. It also often gives prompt relief in the pain of sciatica, lumbago, intercostal neuralgia, zoster and similar conditions if injected in the neighborhood of the affected nerves.

It should not be used in the cardiac depression due to chloroform or ether narcosis, inasmuch as it is likely to intensify the loss of heart power in these cases rather than relieve it.

Ether should be injected with a glass or metallic syringe, it being destructive to rubber or celluloid. The syringe should always be properly oiled after making an injection, as the oil on the piston is dissolved by the ether. The dose to be injected is from ten to sixty minims, the most frequent dose being fifteen minims. Some smarting usually attends the injection but soon disappears. The injection is followed by a slight puffy swelling, which, ordinarily, soon subsides. It has been injected three or four times a day in adynamic pneumonia. When sudden extreme depression of the heart is to be overcome, ten or twenty minims may be injected every five minutes until some result is reached.

The curative results of the subcutaneous use of ether are not only different in degree, but in kind, from the stomachal administration of the same agent.

